# Predictors of mortality in hospitalized patients with disseminated histoplasmosis

**DOI:** 10.1016/j.bjid.2026.105891

**Published:** 2026-07-07

**Authors:** Francielly M. Gastaldi, Franciny M. Gastaldi, Alessandro C. Pasqualotto

**Affiliations:** a Hospital de Clínicas de Uberlândia – Ebserh, Uberlândia, MG, Brazil; b Missão Sal da Terra, Uberlândia, MG, Brazil; c Universidade Federal de Ciências da Saúde de Porto Alegre (UFCSPA), Porto Alegre, RS, Brazil; d Santa Casa de Porto Alegre, Porto Alegre, RS, Brazil

**Keywords:** AIDS, Histoplasmosis, HIV, Opportunistic infections, Mortality predictors

## Abstract

**Introduction:**

Histoplasmosis is an important opportunistic infection among People Living with HIV (PLHIV) and still presents significant mortality. Reliable prognostic markers are essential to guide early management and appropriate clinical decision-making. This study aimed to evaluate predictors of death at admission among patients with disseminated histoplasmosis.

**Methods:**

This was a single-center retrospective cohort study including adults with HIV and disseminated histoplasmosis admitted to a Brazilian tertiary hospital between 2009 and 2023. The primary outcome was in-hospital mortality. Variables collected at admission were analyzed using univariate and multivariate approaches.

**Results:**

The study included 105 adults, with a median age of 39-years, predominantly male (82.8%), and presenting severe immunosuppression (median CD4+ T-cell count of 63 cells/µL). Thirty patients (28.5%) died during hospitalization. Elevated urea, ferritin, and Lactate Dehydrogenase (LDH) levels were associated with higher mortality. Coinfections, including pneumocystosis and mycobacterial infections, showed no statistically significant association with the outcome. Multivariate analysis identified tachypnea as the only independent predictor of mortality (OR = 3.9; 95% CI 1.39–10.97; *p* < 0.001), whereas dysphagia remained a protective factor (OR = 0.18; 95% CI 0.03–0.98; *p* = 0.047).

**Conclusions:**

Tachypnea at admission was the only independent predictor of in-hospital mortality. Dysphagia may promote earlier healthcare-seeking behavior, potentially contributing to lower mortality. These findings represent noninvasive and low-cost measures that may be useful for early risk stratification and resource optimization in resource-limited endemic settings.

## Introduction

Histoplasmosis is an opportunistic infection associated with high mortality among People Living with HIV/AIDS (PLHIV), particularly in endemic regions.^1^, ^2^, ^3^, ^4^ In the Americas, Africa, and Asia, it represents an important differential diagnosis in the clinical evaluation of suspected mycobacterial infections.^2^^,^^3^^,^^5^

Particularly in Brazil, there is considerable variability in the incidence of this fungal infection across different regions, with higher rates observed in the Midwest (Goiás State) and Northeast regions, especially Ceará State.^5^^,^^6^

Although diagnostic tests, such as Histoplasma urinary antigen detection assays, are available, their limited accessibility in most public health systems contributes to underdiagnosis and the persistence of high mortality rates.^4^, ^5^, ^6^, ^7^

Despite appropriate antifungal therapy, mortality among PLHIV with disseminated histoplasmosis remains between 30% and 45%, depending on available resources and the region analyzed.^4^^,^^7^ The initiation and sustained adherence to Antiretroviral Therapy (ART), leading to viral suppression and immune recovery, are also essential to improve prognosis.^5^^,^^8^, ^9^, ^10^

Although mortality predictors are well established for other opportunistic infections in PLHIV, such as pneumocystosis and cryptococcosis, evidence regarding histoplasmosis remains limited.^8^^,^^11^ Variables associated with poor prognosis in histoplasmosis include hypotension, renal dysfunction, and high fungal burden; however, these observations still lack robust validation.^8^^,^^10^^,^^12^ Identifying reliable prognostic markers is essential to guide clinical management.^8^^,^^9^^,^^13^^,^^14^ Simple, low-cost, and widely applicable indicators may facilitate early risk stratification, enabling closer monitoring and timely interventions in high-risk patients.^5^^,^^11^, ^12^, ^13^

This study aimed to identify admission predictors of in-hospital mortality among PLHIV hospitalized with histoplasmosis.

## Methods

This retrospective single-center cohort study was conducted at a tertiary care hospital located in a histoplasmosis-endemic region (Uberlandia, Brazil). The study period extended from January 2009 to December 2023, encompassing 15-years of data collection.

Eligible participants were adults (≥ 18-years) with confirmed HIV infection (diagnosed prior to or during the index hospitalization), admitted to the hospital during the study period, and with laboratory-confirmed histoplasmosis. Diagnostic methods available at the institution included fungal culture (with buffy coat specimens being the most frequently used material for diagnosis) and/or histopathological examination. Antigen detection assays were not available at the institution during the study period, although this method was available through the Brazilian Ministry of Health’s “Advanced AIDS Rapid Care Pathway” strategy (*Circuito Rápido de Aids Avançada*). Exclusion criteria included absence of confirmed fungal infection, incomplete medical records, or loss to follow-up during hospitalization (e.g., transfer to another institution or self-discharge).

Variables of interest were extracted from medical records corresponding to the first day of hospitalization, regardless of the care setting (emergency department, general ward, or intensive care unit). Demographic data included age and sex, as well as HIV-related information such as time between admission and HIV diagnosis, history of Antiretroviral Therapy (ART) use, CD4+ T-cell count, and HIV viral load. All patients originated from the *Triângulo Mineiro* region. Other epidemiological data, such as animal exposure and/or rural residence, were not documented in the medical records. Social vulnerability was not explicitly described in the records, although illicit drug use was frequently reported in the analyzed population.

Clinical variables included fever, weight loss (percentage relative to previous body weight), cough, dyspnea, altered mental status, gastrointestinal symptoms, and hemorrhagic manifestations. Physical examination findings included hepatomegaly, splenomegaly, tachypnea, tachycardia, lymphadenopathy, and mucocutaneous lesions. Laboratory parameters included complete blood count, renal function and electrolytes, coagulation profile, liver function tests and enzymes, Lactate Dehydrogenase (LDH), and ferritin levels. Imaging studies (e.g., chest radiography and ultrasonography) were also reviewed.

The primary outcome of the study was overall in-hospital mortality. For statistical analysis, survivors and non-survivors were compared using the Chi-Square test or Fisher’s exact test for categorical variables, and Student’s *t*-test or Mann-Whitney *U test* for continuous variables, according to data distribution. Variables with a p-value < 0.10 in univariate analysis were entered into multivariate logistic regression, based on Multivariate Analysis of Variance (MANOVA), to identify independent predictors. Odds Ratios (OR) with 95% Confidence Intervals (95% CI) were reported. Model calibration was assessed using the Hosmer-Lemeshow test, and model performance was evaluated using pseudo-*R^2^*. Two-sided p-values < 0.05 were considered statistically significant. All analyses were performed using SPSS (version 30.0.0.0) and JASP (version 0.95.1.0).

The study was approved by the Research Ethics Committee of the Federal University of Uberlandia (CAAE n° 80,534,124.3.0000.5152). Given its retrospective design, the Committee granted a waiver of written informed consent, as no direct contact or intervention with the study population was involved.

## Results

During the study period, a total of 105 patients were included. The median age was 39 years (Interquartile Range [IQR], 19–72 years), with a predominance of males (82.8%). At baseline, the median HIV viral load was 625,257 copies/Ml (5.8 log), and the median CD4+ T-cell count was 63 cells/µL. Among those with a previous HIV diagnosis (66/105; 62.8%), only 8-patients (12.1%) were receiving Antiretroviral Therapy (ART) at hospital admission, all for ≤ 3-months; the remaining 58 patients (87.9%) had discontinued treatment.

Histoplasmosis was confirmed mainly by fungal culture from tissue fragments (58/105), bone marrow aspirates (57/105), buffy coat specimens (44/105), or respiratory secretions (2/105). Histopathological examination established the diagnosis in only two cases (1.9%). In most cases, multiple biological specimens were collected for diagnostic evaluation.

The most frequent clinical manifestations were fever (91.4%), weight loss (87.6%; mean loss of 19.6% of body weight), cough (64.7%), dyspnea (51.4%), and tachypnea (44.7%). Gastrointestinal involvement was less prevalent. Among these manifestations, the most frequently reported symptoms were nausea/vomiting (46.6%), diarrhea (44.7%), and dysphagia (23.8%), the latter being more common among patients who survived hospitalization. Laboratory abnormalities included anemia (90.4%) and elevated Lactate Dehydrogenase (LDH) levels (71.4%). Creatinine levels ≥2-times the upper limit of normal at admission were documented in 12.4% of patients.

Regarding antifungal treatment, all survivors received antifungal therapy: itraconazole monotherapy in 7 cases (9.3%); amphotericin B deoxycholate in 49/75 (65.3%); lipid complex formulations in 19/75 (25.3%); and liposomal amphotericin B in 7/75 (9.3%). Among non-survivors, three patients (10%) did not receive antifungal therapy due to death before diagnostic confirmation. Among the remaining patients, amphotericin B deoxycholate was administered in 16/30 (53.3%) and liposomal amphotericin B in 11/30 (36.7%).

Amphotericin B deoxycholate was the most commonly used treatment modality due to its easier availability, either because of immediate institutional access or delayed diagnostic confirmation, which limited requests for lipid formulations. In the univariate analysis, no statistically significant association was found between amphotericin B deoxycholate use and unfavorable outcomes (OR = 0.61; 95% CI 0.26–1.43; *p* ≈ 0.25).

Overall, 30 patients died during hospitalization (mortality rate 28.5%). Survivors (*n* = 75) and non-survivors (*n* = 30) had similar ages (mean 39.2 vs. 39.7 years), male predominance (83.3%vs. 82.6%), and duration of symptoms before admission (3.0 vs. 2.9 months, respectively). However, previous HIV/AIDS diagnosis was more frequent among survivors (80%) than among non-survivors (56%).

Table 1 presents the distribution of histoplasmosis cases according to survival status (survivors vs. non-survivors), as well as admission laboratory findings. Variables significantly associated with mortality in the univariate analysis included hypoalbuminemia (*p* < 0.001), elevated urea (*p* < 0.001), ferritin (*p* = 0.01), and Lactate Dehydrogenase (LDH) levels (*p* = 0.018), as well as altered INR (*p* = 0.015) and reduced hematological parameters (hemoglobin, *p* < 0.01; hematocrit, *p* < 0.001).

**Table 1:** Comparison of laboratory findings at admission between survivors and non-survivors with AIDS-related histoplasmosis.

	All patients (*n* = 105)	Survivors (*n* = 75)	Non-survivors (*n* = 30)	p-value
Hemoglobin (g/dL)	9.4 (4.7‒15.7)	9.9 (4.7‒15.7)	8.2 (5.6‒11.2)	0.0001
Hematocrit (%)	27.8 (14.7‒43.9)	29.2 (14.7‒43.9)	24.5 (15.8‒36.6)	0.0003
White blood cell Count (cel/mm^3^)	3610 (100‒26,500)	3370 (300‒11,600)	4190 (100‒26,500)	0.620
Platelets (cel/mm^3^)	161,790 (4000‒580,000)	173,680 (21,000‒580,000)	132,070 (4000 – 444,000)	0.086
Urea (mg/dL)	35.5 (9‒211.6)	30.5 (9‒96)	62.4 (16.1‒211.6)	0.004
Creatinine (mg/dL)	1.1 (0.4‒7.8)	0.8 (0.4‒2.1)	1.8 (0.4‒7.8)	0.314
International Normalized Ratio	1.3 (1‒15.5)	1.1 (1‒1.9)	1.8 (1‒15.5)	0.015
Aspartate transaminase (U/L)	136.4 (8‒1473)	114.8 (8‒629.5)	190.5 (11‒1473)	0.144
Alanine Transaminase (U/L)	52.6 (5‒242)	50.3 (5‒218)	58.4 (6‒242)	0.540
Gamma-Glutamyltransferase (U/L)	270.8 (29‒1592.7)	295.1 (29‒1592.7)	211.5 (40‒947.4)	0.760
Alkaline Phosphatase U/L)	302 (37‒1330)	290.4 (37‒1330)	330.6 (55‒822)	0.190
Lactate Dehydrogenase (U/L)	1372.7 (126‒7734)	1199.4 (126‒7734)	1776.9 (133‒2380)	0.018
Ferritin (µg/L)	22,418.6 (135‒128,198)	17,238.9 (135‒100,000)	34,677.1 (250.2‒128,198)	0.011
Albumin (g/dL)	2.7 (1.4‒4.7)	2.9 (1.5‒4.7)	2.2 (1.4‒3.5)	0.00001
Total bilirubin (mg/dL)	0.9 (0.1‒8.9)	0.7 (0.1‒7.9)	1.3 (0.1‒8.9)	0.5
Direct bilirubin (mg/dL)	0.6 (0.0‒7.3)	0.4 (0.0‒3.7)	0.9 (0.0‒7.3)	0.28
Indirect (mg/dL) bilirubin	0.3 (0.0‒6.6)	0.3 (0.0‒6.6)	0.3 (0.0‒2.6)	0.8
HIV viral load (copies/mL)	625,009 (32‒10,020,113)	354,905 (32‒2523,965)	1355,625 (77‒10,020,113)	0,16
CD4 T lymphocyte dosage (cel/mm^3^)	63.3 (3‒301)	62.3 (3‒301)	66.3 (6‒258)	0.20
Buffy coat with Histoplasma	44 (41.9%)	28 (37.3%)	16 (53.3%)	0.2
Bone marrow aspiration with Histoplasma	57 (54.3%)	41 (54.6%)	16 (53.3%)	1
Respiratory secretions with Histoplasma	2 (1.9%)	1 (1.3%)	1 (1.3%)	0.5
Tissue fragments with Histoplasma	58 (55.2%)	51 (68%)	7 (23.3%)	<0.001
Skin with Histoplasma	23 (21.9%)	20 (26.6%)	3 (10%)	0.07
Lymph node with Histoplasma	6 (5.7%)	4 (5.3%)	2 (6.6%)	1
Liver with Histoplasma	6 (5.7%)	4 (5.3%)	2 (6.6%)	0.03
Oral mucosa with Histoplasma	6 (5.7%)	5 (6.6%)	1 (3.3%)	0.6
Nasal mucosa with Histoplasma	6 (5.7%)	6 (8%)	0 (0%)	0.2

Overall, no consistent pattern was observed in mortality rates throughout the study period. Higher mortality rates were recorded in 2014 (50.0%), 2020 (42.9%), 2022 (100%), and 2023 (44.4%), whereas no deaths occurred in 2015 and 2018. The year 2009 had the highest number of patients (*n* = 17) and four deaths (23.5%). In subsequent years, the annual number of cases fluctuated substantially, ranging from 2 to 12 patients per year. These oscillations likely reflect sampling variability due to the small number of patients in certain years rather than systematic changes in disease severity or treatment practices.

Table 2 presents the univariate analysis of mortality predictors. Dysphagia (*p* < 0.01) and mucosal lesions (*p* = 0.012) were associated with higher survival rates, whereas tachypnea (*p* < 0.01), tachycardia (*p* < 0.01), and gastrointestinal bleeding (*p* = 0.027) were associated with higher mortality. The analyses demonstrated the association between tachypnea and mortality through multivariate analysis (MANOVA).

**Table 2:** Univariate analysis comparing survivors and non-survivors in AIDS-related histoplasmosis.

Variable	Survivors (*n* = 75)	Non-survivors (*n* = 30)	p-value
Diarrhea	38 (51%)	18 (60%)	0.39
Dysphagia	29 (39%)	2 (6.7%)	<0.01
Dyspnea	36 (48%)	18 (60%)	0.27
Abdominal pain	29 (39%)	10 (33%)	0.61
Splenomegaly	47 (67%)	20 (67%)	0.70
Hemoptysis	4 (5.3%)	2 (6.7%)	1
Hepatomegaly	65 (87%)	25 (83%)	0.76
Hypotension	8 (11%)	4 (13%)	0.74
Skin lesions	44 (59%)	16 (53%)	0.62
Mucosal lesions	32 (43%)	5 (17%)	0.012
Lymphadenopathy	25 (33%)	5 (17%)	0.088
Nausea/vomiting	37 (49%)	19 (63%)	0.19
Gastrointestinal bleeding	9 (12%)	9 (30%)	0.027
Other hemorrhagic events	4 (6.5%)	1 (3.3%)	1
Sweating	16 (21%)	9 (30%)	0.35
Tachycardia	24 (32%)	19 (63%)	< 0.01
Tachypnea	23 (31%)	22 (73%)	< 0.001
Cough	50 (67%)	18 (60%)	0.52

Independent risk factors for mortality are shown in Table 3. After adjustment for confounders, tachypnea at admission remained independently associated with mortality (*p* = 0.013), whereas dysphagia showed a protective effect (*p* = 0.026).

**Table 3:** Multivariate analysis of independent predictors of in-hospital mortality among AIDS patients with histoplasmosis.

Variable	Odds Ratio [95% CI]	p-value
Tachypnea	3.9 [1.39‒10.97]	0.0098
Tachycardia	2.16 [0.78‒5.91]	0.134
Gastrointestinal bleeding	1.58 [0.50‒5.02]	0.43
Mucosal lesions	0.83 [0.21‒3.18]	0.79
Dysphagia	0.18 [0.03‒0.98]	0.047

CI, Confidence Interval.

Table 4 summarizes the frequency of opportunistic infections across groups. Herpes co-infection was significantly more frequent among non-survivors. Regarding mycobacterial infections, five cases were attributed to *Mycobacterium tuberculosis*, whereas four involved unspecified rapidly growing mycobacteria.

**Table 4:** Prevalence of co-infections in the studied populations (survivors and non-survivors).

Co-infeccion	Survivors (*n* = 75)	Non-survivors (*n* = 30)	p-value
Mycobacterial infection	7 (9.3%)	2 (6.6%)	0.002
Pneumocystosis	10 (13.3%)	17 (56.6%)	0.3
Intestinal parasites	7 (9.3%)	3 (10%)	0.005
Herpes infection	17 (22.6%)	3 (10%)	<0.001
Cytomegalovirus infection	6 (8%)	1 (3.3%)	0.002
Oroesophageal candidiasis	7 (9.3%)	4 (13.3%)	0.01
Neurotoxoplasmosis	3 (4%)	2 (6.6%)	0.1
Paracoccidioidomycosis	2 (2.6%)	0	0.08
Cryptococcosis	0	4 (13.3%)	0.5

## Discussion

In this retrospective cohort of PLHIV hospitalized between 2009 and 2023 with AIDS-related histoplasmosis, tachypnea at admission emerged as the only independent predictor of in-hospital mortality, emphasizing the prognostic value of a simple and widely applicable bedside parameter.^5^^,^^12^, ^13^, ^14^ This finding likely reflects severe pulmonary involvement, hypoxemia, and systemic inflammatory response at presentation ‒ features typically associated with advanced disease and delayed diagnosis in this population.

Marked immunosuppression characterized this cohort, with a median CD4+ T-cell count of 66 cells/µL. The high frequency of Antiretroviral Therapy (ART) discontinuation before admission further underscores the importance of sustained viral suppression in preventing histoplasmosis.^11^^,^^14^, ^15^, ^16^, ^17^, ^18^, ^19^ The predominance of male patients and the age profile observed were consistent with previous retrospective studies,^15^, ^16^, ^17^, ^18^ suggesting similar demographic patterns across endemic settings. Despite the wider availability of ART, histoplasmosis remains an important contributor to HIV diagnosis, frequently representing the first recognized opportunistic infection. In the present study, HIV diagnosis during hospitalization for histoplasmosis occurred in 62.8% of cases, slightly exceeding proportions previously reported in Brazil and the United States.^20^, ^21^, ^22^, ^23^

Previous studies have associated mortality in histoplasmosis with factors such as hypotension, renal dysfunction, and fungal burden;[9,12,19,23] however, these findings remain inconsistent and are often limited by the lack of multivariate adjustment. In contrast, tachypnea may represent a more direct clinical marker of extensive pulmonary involvement, whether resulting from fungal infiltration itself or concomitant opportunistic infections.^19^^,^^22^^,^^23^

Although previous reviews have described stronger associations between histoplasmosis and mycobacterial infections, with co-infection rates ranging from 10% to 20%, the frequency observed in the present cohort was lower (8.6%; 9/105). Conversely, the higher prevalence of pneumocystosis suggests greater pulmonary involvement, supporting the clinical relevance of tachypnea despite the potential confounding effect related to the intrinsic mortality associated with other opportunistic infections. Nevertheless, this pattern was not accompanied by significant differences in mortality rates. These findings may reflect regional epidemiological characteristics or diagnostic limitations, highlighting the need for further investigation.^22^, ^23^, ^24^, ^25^

Regarding antifungal therapy, amphotericin B deoxycholate was the most frequently prescribed treatment. This pattern likely reflects the limited availability of lipid formulations in some centers, influenced by both restricted awareness of existing dispensing pathways and diagnostic delays resulting from laboratory limitations. Such barriers may postpone diagnostic confirmation and consequently delay requests for lipid formulations, despite the existence of Brazilian Ministry of Health programs aimed at providing these medications.

Although amphotericin B deoxycholate is associated with greater nephrotoxicity and other adverse events that may negatively affect clinical outcomes, no significant association was found between its use and in-hospital mortality. Accordingly, the therapeutic modality itself was not associated with prognosis in the present study, despite robust evidence supporting superior efficacy and improved safety profiles of lipid formulations in disseminated histoplasmosis. This finding should nevertheless be interpreted cautiously, given the possibility of indication bias and differences in baseline clinical severity that may have influenced treatment selection. Furthermore, deaths occurring before diagnostic confirmation and antifungal initiation may also have contributed to the absence of an observed association.^2^^,^^3^^,^^6^^,^^7^^,^^20^^,^^21^^,^^27^

The identification of an easily accessible clinical marker for risk stratification is particularly relevant in resource-limited endemic settings. Although laboratory assessment remains essential, early recognition of tachypnea may facilitate standardized triage at hospital admission.^1^^,^^9^^,^^12^ Mortality rates varied irregularly throughout the study period without a consistent temporal trend, and the limited number of cases per year may have reduced statistical power for longitudinal comparisons. Given that respiratory rate assessment is simple, inexpensive, and noninvasive, its incorporation into initial evaluation protocols may support earlier identification of high-risk patients, closer monitoring, prioritization of diagnostic resources, and timely therapeutic interventions.

Considering the high burden of histoplasmosis and the heterogeneity of healthcare settings, implementing simple and broadly applicable tools is highly desirable to facilitate routine clinical use.^19^^,^^22^^,^^26^ Such approaches may also complement laboratory evaluation by identifying clinically relevant variables during the initial assessment that could influence management decisions.

Consistent with previous reports, hypoalbuminemia, hematological abnormalities (particularly anemia), renal dysfunction, coagulation disturbances assessed by INR, and elevated ferritin levels were associated with poorer prognosis.^19^^,^^24^, ^25^, ^26^, ^27^, ^28^ and reached statistical significance in the present analysis. These findings reinforce the potential value of targeted laboratory assessment to support early clinical decision-making, especially in resource-constrained settings. Nevertheless, due to limitations such as small sample sizes and data variability, these associations have not been consistently reproduced across cohorts,^26^^,^^29^^,^^30^ emphasizing the need for further validation.

The relationship between dysphagia and lower mortality has been scarcely addressed in the literature. Available studies.^10^^,^^12^^,^^14^^,^^17^^,^^27^^,^^31^ suggest that gastrointestinal manifestations ‒ particularly dysphagia ‒ may prompt earlier healthcare seeking, thereby facilitating timely clinical evaluation and diagnostic investigation. In the present study, dysphagia behaved as a protective factor in both univariate and multivariate analyses, reinforcing its independent association with lower mortality. By contrast, other gastrointestinal manifestations, including diarrhea, nausea/vomiting, and abdominal pain, showed no significant relationship with mortality, suggesting that these symptoms may represent nonspecific manifestations of gastrointestinal involvement without independent prognostic value.

The higher frequency of gastrointestinal bleeding among non-survivors should be interpreted cautiously. This finding may reflect more extensive mucosal involvement, coagulopathies, or associated organ dysfunction, potentially serving as an indirect marker of disseminated disease rather than an independent determinant of mortality. Accordingly, no direct statistically significant relationship was observed between gastrointestinal bleeding and the evaluated outcome.

Several hypotheses may explain the apparent protective role of dysphagia. The symptom may occur in patients with more extensive mucosal involvement or may generate greater discomfort and functional impairment compared with respiratory manifestations, leading patients to seek medical care earlier. Such earlier presentation could contribute to more favorable outcomes. However, these interpretations remain speculative and require confirmation in larger studies with robust methodological designs capable of clarifying the behavioral and pathophysiological mechanisms underlying this association.

Although these findings were derived from a retrospective analysis, the clinical characteristics of this population closely resemble those reported in previous studies, supporting their potential reproducibility. Moreover, considering the frequent occurrence of co-infections ‒ which have not consistently demonstrated associations with mortality[26,28,30] — the presence of other opportunistic infections would not necessarily preclude the clinical applicability of this criterion.

This study was conducted in an endemic region but remains limited by its retrospective and single-center design. Prospective multicenter studies are warranted to validate tachypnea as a prognostic marker and determine whether targeted interventions may reduce mortality in this population. Another limitation is the absence of Histoplasma antigen detection testing, which may restrict the generalizability of these findings to settings where earlier diagnosis is feasible and prompt treatment initiation contributes to lower mortality.

## Conclusion

In this retrospective cohort of People Living with HIV (PLHIV) and disseminated histoplasmosis, tachypnea at admission was the only independent predictor of in-hospital mortality. As a simple and readily available clinical sign, respiratory rate may be incorporated as an early risk stratification tool, contributing to the optimization of clinical management and allocation of diagnostic and therapeutic resources in this vulnerable population.

Additionally, the independent association between dysphagia and improved survival should be interpreted with caution. Based on the present findings, this association may be related to earlier healthcare-seeking behavior. However, further studies with more robust designs are needed to better understand this relationship.

Laboratory abnormalities also demonstrated prognostic relevance. Hypoalbuminemia, anemia, renal dysfunction, coagulation abnormalities assessed by INR, and hyperferritinemia were associated with poorer outcomes, reinforcing the importance of initial laboratory evaluation in suspected or confirmed disseminated histoplasmosis for clinical severity stratification.

Despite the limitations inherent to the retrospective design, the single-center setting, and the unavailability of Histoplasma antigen detection, the findings highlight the potential utility of accessible clinical parameters for optimizing initial management. Prospective multicenter studies are warranted to validate these findings and determine their impact on mortality reduction.

## Conflicts of interest

The authors declare no conflicts of interest.

## Data Availability

The data that support the findings of this study are available from the corresponding author upon reasonable request.
